# Supplementary material for: Caregiver acceptability of the guidelines for managing young infants with possible serious bacterial infections (PSBI) in primary care facilities in rural Bangladesh
Source: PLoS One. 2020 Apr 14;15(4):e0231490. doi: 10.1371/journal.pone.0231490 (PMC7156040; doi:10.1371/journal.pone.0231490)
Supplement: S1 Table — (PDF) [file pone.0231490.s001.pdf]

**Levels of influence**	**Description**	**Constructs explored in the interviews**
Individual	Factors related to the caregivers' knowledge and perception of care provided per the guidelines including antibiotics and referral	Caregiver autonomy in decision-making to seek care and accept referral; caregiver perception of illness severity; previous experiences with care at public sector facilities
Interpersonal	The role and influence household factors and other key actors in healthcare decision-making	Influence of economic factors, household responsibilities, and family members in decision-making for the infant's care (e.g., husband, mother or mother-in-law, other family members)
Organizational	Care received at public sector health facility and relationship with providers	Availability and accessibility of public sector services and medicines; Caregiver relationship with the provider; Provider motivation to facilitate referral and follow-up
Community	Social and cultural values that motivate or impede acceptability of treatment per the guidelines	Social and cultural values that influence care-seeking in this context; community perceptions of public sector care
