# Supplementary material for: Caregiver acceptability of the guidelines for managing young infants with possible serious bacterial infections (PSBI) in primary care facilities in rural Bangladesh
Source: PLoS One. 2020 Apr 14;15(4):e0231490. doi: 10.1371/journal.pone.0231490 (PMC7156040; doi:10.1371/journal.pone.0231490)
Supplement: S1 File — (PDF) [file pone.0231490.s002.pdf]

In Depth Interview (IDI) Guide:
Health Providers (SACMO)

IRB No.: 6607
PI: Abdullah Baqui
PI Version: 2; Date: 28 March 2016

General instructions: Interviewers, please read the following opening script (Part B) to the respondent and then proceed with asking him/her the questions listed in Part C. Some questions have specific probes, but please feel free to probe on additional points of interest to guide the discussion as needed.

Part A: Background Information

**1. IDI Code:**			**2. Interviewer Code:**		
**3. Date:**		**4. Start Time:**		**5. End time:**	
**6. Location:**			**7. FWC where SACMO works:**		
**8. Description of Setting (Surroundings, general atmosphere, weather, observations before starting IDI)**					

Part B: Opening Scripts

Opening script—Individual interview with SACMO: Thank you for agreeing to participate in our study. As previously mentioned, we are going to ask you some questions about your experience as a SACMO implementing the new guidelines for infection management in young infants. Please respond to these questions as honestly and descriptively as possible. Your identity will be kept confidential so please feel comfortable sharing your stories. We will now begin!

Part C: Guiding Questions

Case management
1. Please describe your management process in general of the infection of young infants. How do you use the new guidelines when treating these infants?
2. What challenges do you encounter in implementing the new guidelines?
    - Assessing cases?
    - Referring cases?
    - Treating cases?
3. What are the families' reactions to the treatment guidelines (referral and/or home treatment)?
    - Can you tell me about a specific example of how they reacted that way?
4. If there are any, what challenges to implementing the new guidelines exist?
    - How should they be addressed?
5. How distant is your home from this center? What are the implications, if any, of this distance on your management of infection on young infants?

6. Do you manage young infants with signs of infection differently in your private practice than what is described in these guidelines? (Probe: What are the differences? Why do you do it differently?)
7. What happens in case management, referral and treatment of young infant with signs of infection in case you are absent in the center?
8. How are you held accountable for conducting follow-up visits with young infant infection cases? (Probe: Day 2 and Day 4 follow up)

Supervision

9. Please tell me about the supervision visits you received related to management of infection of young infants? What happened during these visits?
10. How would you describe the effectiveness of these supervision visits?
    - Probe: Did you find the supervision visit to be helpful? Please explain.
11. What were your expectations of these supervision visits?
    - How did these supervision visits meet your expectations?
      What do you wish happened during these supervision visits that did not happen? Please explain.

Monitoring

12. What is the process for completing reports on the implementation of the new guidelines?
    a. What is your opinion about the reporting format? (Probe: What are the good things? Things that need improvement?)
    b. What changes would you like to see to the report, if any? (Probe: Format, frequency, content)
13. Please describe the feedback you have received on your reports if you have received any.

Supply chain

14. How are the UH&FWCs supplied with drugs for the new guidelines? (Probe: How does this compare to other drugs supplied to UH&FWC?)
15. What has been the status of the drug supply? Have there been any issues? (Probe: any stock outs?) What are the reasons?
16. What changes do you think should be made to the drug supply systems, if any?
17. Have there been any changes recently in terms of drug supply system? (Probe: Is it still supplied by Projahnmo or MoHFW is gradually taking over? In sign of change in this light?)

Other issues

18. Do you want to add anything else which we might have missed? (Probe: any suggestions, off the record complaints, off the record challenges, anything else)
