# Supplementary material for: Caregiver acceptability of the guidelines for managing young infants with possible serious bacterial infections (PSBI) in primary care facilities in rural Bangladesh
Source: PLoS One. 2020 Apr 14;15(4):e0231490. doi: 10.1371/journal.pone.0231490 (PMC7156040; doi:10.1371/journal.pone.0231490)
Supplement: S2 File — (PDF) [file pone.0231490.s003.pdf]

Focus Group Discussion Guide:
Health Provider (SACMO)

IRB No.: 6607
PI: Abdullah Baqui
PI Version: 2; Date: 19 March 2016

General instructions: This guide provides the questions and topics that will be addressed in the FGD. The guiding questions are listed by subheadings ‡‡related to program processes. Please adhere to the following instructions when facilitating this FGD:

1. Please begin by welcoming individuals as they arrive to the FGD and obtaining *consent*
2. Fill out **Part A: Background Information** after obtaining consent and before starting the FGD.
3. When it is time to begin, start the recorder, welcome participants as a group and read **Part B: Opening Script**
4. Proceed with **Part C: Guiding Questions** and facilitate the discussion
5. After the discussion concludes, thank the SACMOs for their participation, stop the recorder, and dismiss the participants. Record the end time on the **Part A: Background Information** table

Part A: Background Information

<table>
<tr><td>1. FGD Code:</td><td colspan="2"></td><td colspan="2">2. Facilitator Code:</td><td colspan="2"></td></tr>
<tr><td>3. Date:</td><td></td><td colspan="2">4. Start Time:</td><td></td><td>5. End time:</td><td></td></tr>
<tr><td>6. Location:</td><td colspan="2"></td><td colspan="2">7. # Of participants:</td><td colspan="2"></td></tr>
<tr><td colspan="3">8. Description of Setting (Surroundings, general atmosphere, weather, observations before starting FGD)</td><td colspan="4"></td></tr>
</table>

<table>
<tr><td colspan="6">8. SACMO Information</td></tr>
<tr><td>Name (Code)</td><td>Gender</td><td>Religion</td><td>Years Working as a SACMO</td><td>Years Working Here</td><td>Do they belong to this area (union/upazila?)</td></tr>
<tr><td></td><td></td><td></td><td></td><td></td><td></td></tr>
<tr><td></td><td></td><td></td><td></td><td></td><td></td></tr>
<tr><td></td><td></td><td></td><td></td><td></td><td></td></tr>
<tr><td></td><td></td><td></td><td></td><td></td><td></td></tr>
<tr><td></td><td></td><td></td><td></td><td></td><td></td></tr>
<tr><td></td><td></td><td></td><td></td><td></td><td></td></tr>
</table>

Part B: Opening Script

Good morning/afternoon, Thank you for taking the time to join this FGD in which we hope to learn more about the new guidelines for managing infections in young infants. I am [name] and my colleagues are [name] and [name]. We are a team of researchers engaged by Johns Hopkins University to conduct a study in collaboration with the MOH in order to help strengthen this program. Our main focus is to hear SACMO opinions about how the guidelines are working and how the program is supported.

In the discussion we will be interested to hear your experiences with the implementing the new guidelines for management of infections in young infants. As such there are no right or wrong answers because you are only expected to share your experiences. This should not be viewed as a job evaluation exercise but rather a contribution to help strengthen the program. The information that you share with us will be combined with the opinions we receive from other participants, and your name will not be reported. The information that we share in this group should be considered confidential. Each of you has been given a number that will be used to record your comments, so you will not be identified by name. Please do not share the opinions of others outside of this group. We should respect each others' opinions and give each other turns when speaking. Please let's begin with everyone introducing themselves using their number, and telling the group how long you have been working in your position, and what you did prior to that.

Part C: Guiding Questions

Training

1. How do you and your SACMO colleagues generally feel about the training they received on the new guidelines?

Case management

2. What are the benefits that you and your colleagues see to using these new treatment guidelines?
3. What are the challenges when using these new guidelines when managing cases? How can these be addressed?
4. What are the families' reactions to the treatment guidelines (referral and/or home treatment)?

Technical Materials

5. Please share your opinion of the technical materials that support you in the implementation of the new guidelines (probe: job aid, register, etc.)

Managing infants with infection in private practice vs. public practice

6. Do you manage young infants with signs of infection differently in your private practice than what is described in these guidelines? (Probe: What are the differences? Why do you do it differently?)

   Have you changed how you manage infant infections in your private practice as a result of this new training? (Probe: If yes, what ‡are the changes? Why or why not?)

Supply chain

7. Describe the availability and supply of drugs for implementing the new guidelines - (Probe: How does this compare to other drugs supplied to UH&FWC?)
8. Have there been any issues with supply? (Probe: any stock outs?) What are the reasons?
9. What changes do you think should be made to the drug supply systems, if any? Have there been any changes recently in terms of drug supply system? (Probe: Is it still supplied by Projahnmo or MoHFW is gradually taking over? In sign of change in this light?)

Community mobilization

10. What opinions do you have about the community mobilization activities?
11. What concerns came up in community mobilization events? How were these addressed?
12. What successes and challenges have you witnessed in this community mobilization approach? How would you improve the challenges faced?
13. What do you think should be done for community mobilization if they are not aware of community mobilization activities?

Other issues

14. How are you held accountable for conducting follow-up visits?
15. How distant is your home from this center? What are the implications, if any, of this distance on your management of infection on young infants?
16. Do you want to add anything else which we might have missed? (Probe: any suggestions, off the record complaints, off the record challenges, anything else)
