# Supplementary material for: Caregiver acceptability of the guidelines for managing young infants with possible serious bacterial infections (PSBI) in primary care facilities in rural Bangladesh
Source: PLoS One. 2020 Apr 14;15(4):e0231490. doi: 10.1371/journal.pone.0231490 (PMC7156040; doi:10.1371/journal.pone.0231490)
Supplement: S3 File — (PDF) [file pone.0231490.s004.pdf]

IRB6607_Guide8_v1_2016.08.30

Follow-up Question Guide:

Health Providers (SACMO)

IRB No.: 6607

PI: Abdullah Baqui

PI Version: 1; Date: 30 August 2016

General instructions: Interviewers, please read the following opening script (Part B) to the respondent and then proceed with asking him/her the questions listed in Part C. Some questions have specific probes, but please feel free to probe on additional points of interest to guide the discussion as needed.

Part A: Background Information

<table>
<tr><td>1. IDI Code:</td><td colspan="2"></td><td colspan="2">2. Interviewer Code:</td><td colspan="2"></td></tr>
<tr><td>3. Date:</td><td></td><td colspan="2">4. Start Time:</td><td></td><td>5. End time:</td><td></td></tr>
<tr><td>6. Location:</td><td colspan="2"></td><td colspan="2">7. Union where FPI works:</td><td colspan="2"></td></tr>
<tr><td colspan="3">8. Description of Setting (Surroundings, general atmosphere, weather, observations before starting IDI)</td><td colspan="4"></td></tr>
</table>

Part B: Opening Scripts

Opening script—Follow-up interview with SACMO: Thank you for agreeing to participate in this brief follow-up interview. As previously mentioned, we are going to ask you some follow-up questions based on some early findings from our study activities. As our previous interviews, these questions will be based on your experience implementing the new guidelines for infection management in young infants. Please respond to these questions as honestly and descriptively as possible. Your identity will be kept confidential so please feel comfortable sharing your stories. We will now begin!

Part C: Guiding Questions

1. Our study findings suggest that caregivers in the community are not aware of the new services available at the UH&FWC for infection management in young infants. In your opinion how can awareness about services be improved in the community?

2. Our study findings reveal that often times, when caregivers are referred to bring their baby to a higher level facility, they do not go. In your opinion what are challenges to families accepting referral to a higher level facility?

3. Our findings indicate that not all infants accept treatment on day 1. Have you had any infection cases that did not receive treatment on day 1 (Probe: injections, oral amoxicillin)?
    a. If yes, why did the infant not receive treatment on the first day?

4. Our findings suggest that some CSI cases do not return on the second day. What are possible reasons for this? In your opinion, why do families not come back on day 2?

5. What do SACMOs recommend the families do when the day 2 visit falls on a non-working day (**Probe**: weekend, day of training)?
    a. How are these special types of situations recorded in the register?

6. Our study findings reveal that some SACMOs request families to return to the UH&FWC on day 4 in lieu of a phone call on this day. Can you please discuss your experience with day 4 follow-up?
    a. Have you requested caregivers to return to the UH&FWC on day 4?
        i. If yes, in your opinion do caregivers comply with the visit on day 4?
    b. What are the caregivers' perceptions of returning on day 4?
    c. What do you do if caregivers do not return on day 4 (**Probe**: phone call, home visit)?

7. After the first year of the implementation of the guidelines, what has been your experience?

8. In your opinion what changes should be made to the program?
