# Supplementary material for: Caregiver acceptability of the guidelines for managing young infants with possible serious bacterial infections (PSBI) in primary care facilities in rural Bangladesh
Source: PLoS One. 2020 Apr 14;15(4):e0231490. doi: 10.1371/journal.pone.0231490 (PMC7156040; doi:10.1371/journal.pone.0231490)
Supplement: S4 File — (PDF) [file pone.0231490.s005.pdf]

In Depth Interview (IDI) Guide:
Caregivers of Cases

IRB No.: 6607
PI: Abdullah Baqui
PI Version: 2; Date: 28 March 2016

General instructions: Interviewers, please read the following opening script (Part B) to the respondent and then proceed with asking him/her the questions listed in Part C. Some questions have specific probes, but please feel free to probe on additional points of interest to guide the discussion as needed.

**1. IDI Code:**			**2. Interviewer Code:**		
**3. Date:**		**4. Start Time:**		**5. End time:**	
**6. Location:**			**7. Relationship of participant to infant:**(*ex: Mother, Aunt, Grandmother*)		
**8. Description of Setting (Surroundings, general atmosphere, weather, observations before starting IDI)**					

Part B: Opening script

Opening script—Individual interview with Caregivers of Young Infant Infection Cases: Thank you for agreeing to participate in our study. As previously mentioned, we are going to ask you some questions about your experience as a caregiver of a young infant that was recently treated for infection. Please respond to these questions as honestly and descriptively as possible. Your identity will be kept confidential so please feel comfortable sharing your stories. We will now begin!

Part C: Guiding Questions

Illness Episode:

1. What are the symptoms that you first observed in your child during the illness you reported?
2. When did you decide to seek care out of the home?
    - What was the reason for seeking care?
    - Who made the decision to seek care?
    - How long after you recognized the first symptoms was this decision made?

Care Seeking:

3. Where did you first seek care? (Probe: self-medication, relatives/neighbors, medicine salesman, village doctor, traditional healer, religious leader, other?)
4. How did you come to the decision to bring your infant to this particular provider (FWC or other type of providers)?
5. How distant is your home from this center? What are the implications, if any, of this distance on your care seeking of your young infant?

Care experience:

6. Describe your visit with the provider (Probe: Ask the following questions about each source of care, and repeat the series of questions until there is no other source of care)
   - How long did you have to wait to see the provider?
   - Who treated your infant while you were there?
   - What did this provider tell you about the baby's illness?
   - What was treatment was given to the baby?
   - Was any injection or medication given?
   - What was the cost to you, if any, for the visit and services provided?

7. Do you visit the private chamber of SACMOS from FWCs? If yes, why? If you have experience of getting your young infant treated in both public and private sector, did you notice any difference? (Probe: What were the differences? Why do you think they did it differently?)

Referral experience

8. Did they make a referral for you to see another provider or hospital?
9. ***If no referral was made:** Please describe the advice that the provider gave to you
   a. **Probe:** Please describe what happened next
10. ***If a referral was made**: Did you accept the referral?
    a. If yes: Describe the process of how you got to the next facility
       i. Transport?
       ii. Cost?
       iii. How long after visit did you go to facility?
       iv. What treatment was given?
    b. If no:
       i. Why was referral refused?
       ii. Describe what happened next (Visiting any other provider)

Day 2 Follow Up Visit

11. After your baby was diagnosed, were you asked to return to the facility on the following day?
    - If no, what follow-up advice were you given?
    - If yes, did you return for this follow-up visit?
      - If you did not return on the next day, then why did you not return for the follow-up visit?
      - If you did return on the next day, can you tell me what happened?
        - What time of day did you arrive? How long did you wait to be seen?
        - Who cared for your infant?
        - What care was provided? What was discussed?
        - What was the cost to you, if any, for the visit and services provided

Treatment Adherence

12. What did you do to treat the sick infant at home?
    - How many days did you treat the infant for?
    - How much medicine did you give each day?
    - How did you decide on that course of treatment?

- o [if treatment was for less than the recommended number of days]: did you decide to stop the treatment early? Why? [if answer is no, explain the number of days that are generally recommended, and ask if they were aware that this was the recommendation]
- Did you discuss treatment with any other family or community members? If so, whom?
- Is there any medicine remaining? Why?
- Would you show me the medicine bottles? (if available)
    - o *Interviewer note: Please make a note of how much medicine if leftover. If possible take a picture of the bottle

Caregiver's self-efficacy about administering medication

13. Who fed the medication to the infant?

14. How did you feel about your ability to administer the medications to your infant properly?
    - If any, what were your reservations about administering the medications to your infant?

15. If any, can you describe the challenges you faced related to administering the medications?
    - Probe: Timing of administering medication?
    - Probe: Cooperation of infant in receiving medication?

16. How did you know that administration of the medication was successful?
    - What helpful strategies were used to administer the medication to your infant?
    - How did you feel after the medication was successfully administered to your infant?

17. What mode of treatment do you prefer- giving medicine to baby at home or taking the medicine at hospital? (Probe: Explore if there is any preference of one mode over the other)
    - Which method of treatment do you prefer? Why?
    - What would make administering the medication to your baby a more positive experience?

At-home Follow Up Visit

18. Did you receive any follow-up visits at home from a health provider?
    - If yes, please describe these visits
        - o Where did they occur?
        - o Who was the provider that made the visit?
        - o How many days after your first visit to the UH&FWC did the visit happen? How many visits did you receive?
        - o What happened during the visit?
    - What did you like about these visits?
    - If anything what would you change about the visit if you had the option?
    - What other follow-up services did you receive? When did you receive these services?
        - o Probe: Did you receive a phone call? Text message?
        - o Please tell me who called you and what was discussed.

19. Can you hold the SACMO accountable if there is any issue regarding the treatment and follow up of your young infant? How?

Treatment Outcome

20. What was the treatment outcome for your baby?
    - Why do you think this was the outcome?
    - Are you satisfied with the care you received from FWCs? Explain.

Other issues

1. Do you want to add anything else which we might have missed? (Probe: any suggestions, off the record complaints, challenges against care seeking, anything else)
