# Supplementary material for: Caregiver acceptability of the guidelines for managing young infants with possible serious bacterial infections (PSBI) in primary care facilities in rural Bangladesh
Source: PLoS One. 2020 Apr 14;15(4):e0231490. doi: 10.1371/journal.pone.0231490 (PMC7156040; doi:10.1371/journal.pone.0231490)
Supplement: S5 File — (PDF) [file pone.0231490.s006.pdf]

Focus Group Discussion Guide:

FGD with Caregivers of Young Infants

IRB No.: 6607
PI: Abdullah Baqui
PI Version: 3; Date: 28 March 2016

General instructions: This guide provides the questions and topics that will be addressed in the FGD. The guiding questions are listed by subheadings related to program processes. Please adhere to the following instructions when facilitating this FGD:

1. Please begin by welcoming individuals as they arrive to the FGD and obtaining *consent*
2. Fill out **Part A: Background Information** after obtaining consent and before starting the FGD.
3. When it is time to begin, start the recorder, welcome participants as a group and read **Part B: Opening Script**
4. Proceed with **Part C: Guiding Questions** and facilitate the discussion
5. After the discussion concludes, thank the caregivers for their participation, stop the recorder, and dismiss the participants. Record the end time on the **Part A: Background Information** table

Part A: Background Information

**1. FGD Code:**			**2. Facilitator Code:**		
**3. Date:**		**4. Start Time:**		**5. End time:**	
**6. Location:**			**7. # Of participants:**		
	**8. Description of Setting (Surroundings, general atmosphere, weather, observations before starting FGD)**				
	8. **Caregiver Information**				

Name (Code)	Age	Gender	Religion	Relationship to child (Mother, grandmother, etc.)

Part B: Opening Scripts

Opening script—Group interview: *Good morning/afternoon, I would like to thank you for giving the time to speak with us in this interview in which we would like to learn more from you about your recent experience seeking care for your sick infant. Firstly, let me introduce myself. I am [name] and my colleagues are [name] and [name]. We are a team of researchers engaged by Johns Hopkins University to conduct a study in collaboration with the Ministry of Health in order to help strengthen this program. As such there are no right or wrong answers because you are only expected to share your experiences. In addition to that, let me say that the information that we share in this group should be considered confidential. Each of you has been given a number that will be used to record your comments, so you will not be identified by name. Please do not share the opinions of others outside of this group. We should respect each others' opinions and give each other turns when speaking.*

Please let's begin with everyone introducing themselves using their number.

Part C: Guiding Questions

Care seeking:

1. How do caregivers of infants in the community know when their baby is sick? What symptoms would they notice?
    - Probe: Anything else?
2. When do caregivers in the community generally decide to seek care out of the home?
    - Probe: Who usually makes the decision to seek care?
3. Where do people usually first seek care when their babies are sick?
    - Why is this the first choice provider?
    - Describe what happens when people go to this provider?
        - Probe: What kind of care is administered?
4. Do people ever seek care from more than one provider?
    - If yes, where? Describe the experience.
    - If yes, why do they see more than one provider?
5. What are the illnesses or issues for which you would go to FWC? Anything else?
    - What are the illnesses for which you would go elsewhere? Where would you go for these illnesses?

Care at the union facility

6. What do caregivers in the community think about the FWCs/Union Level Health Facilities? (Probe: Please make sure they understood which facility you are talking about)
    - What do people like about the FWC?
    - What do people dislike about going to the FWC?
    - What generally happens when someone visits an FWC with their sick baby?
        - Probe: Who cares for them (nurse, doctor, SACMO)?
        - Probe: How long is the wait to be seen?
        - Probe: What care is usually given?
    - What level of satisfaction or dissatisfaction do people have in regard to the care they receive at the FWC?
7. What kinds of referrals, if any, does the FWC usually make?
    - Do people generally follow referrals made by the FWC to other providers?
    - Why? Why not?
8. What other services related to management of young infants do the FWCs provide?
    - What follow-up services are made by the FWC, if any?

9. Do you visit the private chamber of SACMOS from FWCs? If yes, why? If you have experience of getting your young infant treated in both public and private sector, did you notice any difference? (Probe: What were the differences? Why do you think they did it differently?)

Treatment Adherence

10. Describe how caregivers in the community generally feel about administering medication to their infants
    - What, if any, are the concerns they might have about administering medication themselves?

11. How do they learn the directions for administering medication to their infants?
    - Is there anything that could help families know how to administer medications correctly?
    - When would they stop administering medication to the infant?
    - Is there ever any leftover medication? What happens if there are leftovers?

Other issues

1. Do you want to add anything else which we might have missed? (Probe: any suggestions, off the record complaints, challenges against care seeking, anything else)
